# 4-[(*E*)-1-Naphthyl­diazen­yl]phenol

**DOI:** 10.1107/S1600536809009866

**Published:** 2009-03-25

**Authors:** Leonid A. Aslanov, Ksenia A. Paseshnichenko, Alexandr V. Yatsenko

**Affiliations:** a Department of Chemistry, Moscow State University, 119992 Moscow, Russian Federation

## Abstract

The title compound (C. I. Solvent Yellow 8), C_16_H_12_N_2_O, crystallizes with two crystallographically independent mol­ecules in the asymmetric unit. The planarity of both mol­ecules is slightly distorted, the dihedral angles between the benzene ring and the naphthalene system being 9.04 (8) and 5.69 (3)°. In the crystal, O—H⋯N hydrogen bonds between the hydr­oxy groups and azo N atoms link the two symmetry-independent mol­ecules into a polymeric chain propagating in [001].

## Related literature

For the crystal structures of similar azo compounds, see: Alder *et al.* (2001 ▶); Petek *et al.* (2006 ▶). For details of the synthetic procedure, see: Fierz-David & Blangey (1949 ▶).
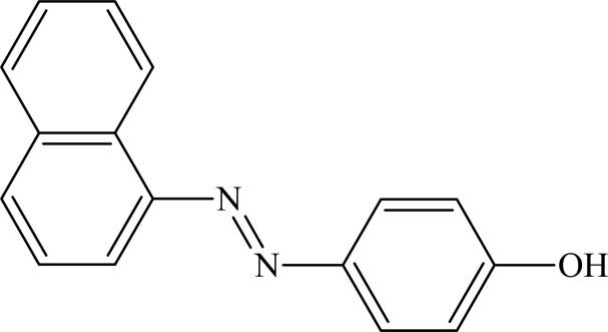


## Experimental

### 

#### Crystal data

C_16_H_12_N_2_O
*M* *_r_* = 248.28
Monoclinic, 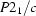

*a* = 10.877 (3) Å
*b* = 19.402 (4) Å
*c* = 13.062 (4) Å
β = 107.91 (2)°
*V* = 2623.0 (12) Å^3^
*Z* = 8
Cu *K*α radiation
μ = 0.64 mm^−1^
*T* = 293 K
0.42 × 0.25 × 0.20 mm

#### Data collection

Enraf–Nonius CAD-4 diffractometer
Absorption correction: none
4759 measured reflections
4759 independent reflections
3656 reflections with *I* > 2σ(*I*)
3 standard reflections frequency: 120 min intensity decay: none

#### Refinement

*R*[*F* ^2^ > 2σ(*F* ^2^)] = 0.035
*wR*(*F* ^2^) = 0.085
*S* = 1.27
4759 reflections
345 parameters
H-atom parameters constrained
Δρ_max_ = 0.12 e Å^−3^
Δρ_min_ = −0.10 e Å^−3^

### 

Data collection: *CAD-4 Software* (Enraf–Nonius, 1989 ▶); cell refinement: *CAD-4 Software* ; data reduction: *PROFIT* (Streltsov & Zavodnik, 1989 ▶) routine of *WinGX* (Farrugia, 1999 ▶); program(s) used to solve structure: *SHELXS97* (Sheldrick, 2008 ▶); program(s) used to refine structure: *SHELXL97* (Sheldrick, 2008 ▶); molecular graphics: *PLATON* (Spek, 2009 ▶); software used to prepare material for publication: *PLATON*.

## Supplementary Material

Crystal structure: contains datablocks global, I. DOI: 10.1107/S1600536809009866/gk2194sup1.cif

Structure factors: contains datablocks I. DOI: 10.1107/S1600536809009866/gk2194Isup2.hkl

Additional supplementary materials:  crystallographic information; 3D view; checkCIF report

## Figures and Tables

**Table 1:** Hydrogen-bond geometry (Å, °)

*D*—H⋯*A*	*D*—H	H⋯*A*	*D*⋯*A*	*D*—H⋯*A*
O1—H1⋯N11	0.82	2.03	2.8380 (15)	167
O11—H11⋯N1^i^	0.82	2.04	2.8485 (15)	170

Symmetry code: (i) 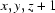.

**Fig. 1.**
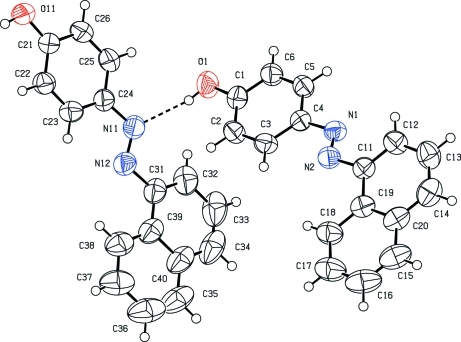
The molecular structure of the title compound with atom labels and 50% probability displacement ellipsoids for non-H atoms.

**Fig. 2.**
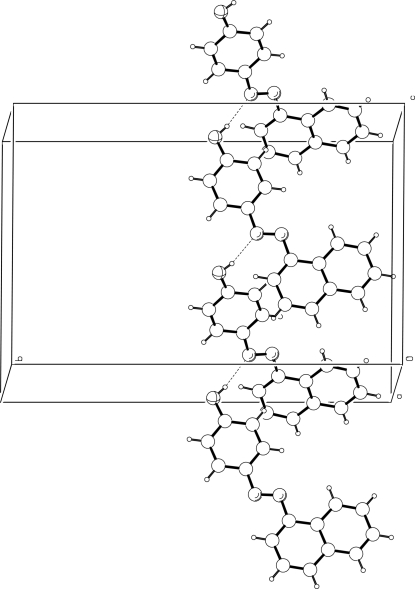
Hydrogen-bonded chain in the structure of the title compound.

## supplementary crystallographic information

### Experimental

The title compound was prepared by coupling of 1-naphthyldiazonium chloride with phenol. For details of the synthetic procedure, see Fierz-David & Blangey (1949). Single crystals were grown by slow evaporation of ethanol solution.

### Refinement

H atoms were located in a difference map and refined freely, but at the final stage they were positioned geometrically and refined using a riding model with C—H = 0.93 Å, O—H = 0.82 Å and with *U*_iso_ (H) = 1.2 times *U*_eq_ (C), *U*_iso_ (H) = 1.5 times *U*_eq_ (O)

### Crystal data

**Table:**

C_16_H_12_N_2_O	*F*(000) = 1040
*M**_r_* = 248.28	*D*_x_ = 1.257 Mg m^−^^3^
Monoclinic, *P*2_1_/*c*	Cu *K*α radiation, λ = 1.54178 Å
Hall symbol: -P 2ybc	Cell parameters from 25 reflections
*a* = 10.877 (3) Å	θ = 30.2–33.6°
*b* = 19.402 (4) Å	µ = 0.64 mm^−^^1^
*c* = 13.062 (4) Å	*T* = 293 K
β = 107.91 (2)°	Prism, yellow
*V* = 2623.0 (12) Å^3^	0.42 × 0.25 × 0.20 mm
*Z* = 8	

### Data collection

**Table:**

Enraf–Nonius CAD-4 diffractometer	*R*_int_ = 0.0000
Radiation source: fine-focus sealed tube	θ_max_ = 68.0°, θ_min_ = 4.2°
graphite	*h* = −13→12
Nonprofiled ω scans	*k* = 0→23
4759 measured reflections	*l* = 0→15
4759 independent reflections	3 standard reflections every 120 min
3656 reflections with *I* > 2σ(*I*)	intensity decay: none

### Refinement

**Table:**

Refinement on *F*^2^	Primary atom site location: structure-invariant direct methods
Least-squares matrix: full	Secondary atom site location: difference Fourier map
*R*[*F*^2^ > 2σ(*F*^2^)] = 0.035	Hydrogen site location: inferred from neighbouring sites
*wR*(*F*^2^) = 0.085	H-atom parameters constrained
*S* = 1.27	*w* = 1/[σ^2^(*F*_o_^2^) + (0.03*P*)^2^] where *P* = (*F*_o_^2^ + 2*F*_c_^2^)/3
4759 reflections	(Δ/σ)_max_ < 0.001
345 parameters	Δρ_max_ = 0.12 e Å^−^^3^
0 restraints	Δρ_min_ = −0.10 e Å^−^^3^

### Special details

**Table:**

Geometry. All e.s.d.'s (except the e.s.d. in the dihedral angle between two l.s. planes) are estimated using the full covariance matrix. The cell e.s.d.'s are taken into account individually in the estimation of e.s.d.'s in distances, angles and torsion angles; correlations between e.s.d.'s in cell parameters are only used when they are defined by crystal symmetry. An approximate (isotropic) treatment of cell e.s.d.'s is used for estimating e.s.d.'s involving l.s. planes.
Refinement. Refinement of *F*^2^ against ALL reflections. The weighted *R*-factor *wR* and goodness of fit *S* are based on *F*^2^, conventional *R*-factors *R* are based on *F*, with *F* set to zero for negative *F*^2^. The threshold expression of *F*^2^ > σ(*F*^2^) is used only for calculating *R*-factors(gt) *etc*. and is not relevant to the choice of reflections for refinement. *R*-factors based on *F*^2^ are statistically about twice as large as those based on *F*, and *R*- factors based on ALL data will be even larger.

### Fractional atomic coordinates and isotropic or equivalent isotropic displacement parameters (Å^2^)

**Table:**

	*x*	*y*	*z*	*U*_iso_*/*U*_eq_	
O1	0.18879 (11)	0.46571 (5)	0.37796 (7)	0.0747 (3)	
H1	0.1683	0.4338	0.4105	0.112*	
N1	0.45502 (10)	0.37955 (5)	0.10200 (8)	0.0539 (3)	
N2	0.50101 (11)	0.31958 (6)	0.11285 (8)	0.0579 (3)	
C1	0.25559 (13)	0.44076 (7)	0.31377 (9)	0.0567 (3)	
C2	0.28294 (13)	0.37149 (7)	0.30773 (10)	0.0600 (3)	
H2	0.2556	0.3396	0.3493	0.072*	
C3	0.35060 (13)	0.34981 (7)	0.24013 (10)	0.0582 (3)	
H3	0.3695	0.3033	0.2363	0.070*	
C4	0.39057 (12)	0.39743 (6)	0.17775 (9)	0.0513 (3)	
C5	0.36279 (13)	0.46612 (7)	0.18396 (10)	0.0589 (3)	
H5	0.3883	0.4979	0.1412	0.071*	
C6	0.29752 (13)	0.48811 (7)	0.25289 (10)	0.0607 (3)	
H6	0.2816	0.5348	0.2585	0.073*	
C11	0.56587 (12)	0.30151 (7)	0.03673 (9)	0.0544 (3)	
C12	0.62216 (14)	0.34891 (7)	−0.01214 (11)	0.0641 (4)	
H12	0.6194	0.3955	0.0039	0.077*	
C13	0.68388 (15)	0.32749 (9)	−0.08621 (12)	0.0761 (4)	
H13	0.7208	0.3599	−0.1203	0.091*	
C14	0.68996 (15)	0.25910 (9)	−0.10836 (12)	0.0767 (4)	
H14	0.7293	0.2455	−0.1590	0.092*	
C15	0.64858 (17)	0.13775 (9)	−0.07262 (14)	0.0855 (5)	
H15	0.6910	0.1231	−0.1206	0.103*	
C16	0.59915 (19)	0.09030 (9)	−0.02100 (16)	0.0989 (6)	
H16	0.6073	0.0436	−0.0337	0.119*	
C17	0.53563 (18)	0.11151 (8)	0.05166 (14)	0.0888 (5)	
H17	0.5013	0.0785	0.0869	0.107*	
C18	0.52309 (15)	0.17902 (7)	0.07175 (11)	0.0699 (4)	
H18	0.4805	0.1920	0.1205	0.084*	
C19	0.57439 (13)	0.23028 (7)	0.01893 (10)	0.0575 (3)	
C20	0.63794 (14)	0.20886 (8)	−0.05609 (11)	0.0659 (4)	
O11	0.37735 (11)	0.47277 (5)	0.92527 (7)	0.0719 (3)	
H11	0.4000	0.4423	0.9706	0.108*	
N11	0.12406 (11)	0.37129 (6)	0.51921 (8)	0.0625 (3)	
N12	0.08804 (11)	0.30945 (7)	0.51060 (8)	0.0641 (3)	
C21	0.31596 (13)	0.44433 (7)	0.82836 (10)	0.0568 (3)	
C22	0.29846 (13)	0.37419 (7)	0.81230 (10)	0.0588 (3)	
H22	0.3302	0.3441	0.8698	0.071*	
C23	0.23447 (13)	0.34851 (7)	0.71195 (10)	0.0586 (3)	
H23	0.2220	0.3013	0.7018	0.070*	
C24	0.18851 (13)	0.39336 (7)	0.62584 (9)	0.0564 (3)	
C25	0.20741 (15)	0.46323 (8)	0.64187 (10)	0.0712 (4)	
H25	0.1777	0.4933	0.5841	0.085*	
C26	0.27000 (16)	0.48893 (7)	0.74295 (10)	0.0730 (4)	
H26	0.2812	0.5362	0.7535	0.088*	
C31	0.02504 (13)	0.28701 (8)	0.40362 (10)	0.0657 (4)	
C32	−0.02955 (14)	0.33091 (10)	0.32012 (11)	0.0815 (5)	
H32	−0.0284	0.3782	0.3320	0.098*	
C33	−0.08743 (17)	0.30435 (13)	0.21628 (13)	0.1033 (7)	
H33	−0.1228	0.3343	0.1592	0.124*	
C34	−0.09212 (17)	0.23539 (13)	0.19873 (14)	0.1049 (7)	
H34	−0.1303	0.2189	0.1294	0.126*	
C35	−0.04644 (18)	0.11655 (13)	0.26655 (17)	0.1062 (7)	
H35	−0.0853	0.0992	0.1978	0.127*	
C36	0.0031 (2)	0.07283 (12)	0.3486 (2)	0.1144 (7)	
H36	−0.0012	0.0256	0.3360	0.137*	
C37	0.06123 (18)	0.09785 (10)	0.45268 (17)	0.1014 (6)	
H37	0.0944	0.0671	0.5090	0.122*	
C38	0.06965 (15)	0.16661 (9)	0.47226 (14)	0.0815 (5)	
H38	0.1094	0.1824	0.5418	0.098*	
C39	0.01899 (13)	0.21457 (9)	0.38867 (11)	0.0700 (4)	
C40	−0.04044 (15)	0.18807 (11)	0.28312 (13)	0.0851 (5)	

### Atomic displacement parameters (Å^2^)

**Table:**

	*U*^11^	*U*^22^	*U*^33^	*U*^12^	*U*^13^	*U*^23^
O1	0.0950 (8)	0.0714 (7)	0.0727 (6)	0.0091 (6)	0.0481 (6)	0.0031 (5)
N1	0.0556 (6)	0.0481 (6)	0.0588 (6)	−0.0007 (5)	0.0187 (5)	−0.0034 (5)
N2	0.0634 (7)	0.0510 (7)	0.0607 (6)	0.0037 (5)	0.0213 (5)	−0.0004 (5)
C1	0.0590 (8)	0.0618 (9)	0.0520 (6)	0.0035 (7)	0.0207 (6)	0.0006 (6)
C2	0.0631 (8)	0.0568 (8)	0.0641 (7)	−0.0053 (7)	0.0253 (6)	0.0061 (6)
C3	0.0623 (8)	0.0456 (8)	0.0680 (7)	−0.0022 (6)	0.0220 (7)	−0.0019 (6)
C4	0.0548 (7)	0.0496 (8)	0.0504 (6)	−0.0002 (6)	0.0175 (5)	−0.0014 (5)
C5	0.0708 (9)	0.0509 (8)	0.0601 (7)	0.0007 (7)	0.0277 (6)	0.0049 (6)
C6	0.0735 (9)	0.0498 (8)	0.0629 (7)	0.0062 (7)	0.0271 (7)	0.0024 (6)
C11	0.0540 (8)	0.0527 (8)	0.0553 (6)	0.0048 (6)	0.0152 (6)	−0.0004 (6)
C12	0.0628 (9)	0.0582 (9)	0.0753 (8)	0.0044 (7)	0.0271 (7)	0.0032 (7)
C13	0.0730 (10)	0.0827 (12)	0.0831 (9)	0.0056 (9)	0.0393 (8)	0.0097 (8)
C14	0.0645 (10)	0.0946 (13)	0.0763 (9)	0.0135 (9)	0.0294 (8)	−0.0080 (9)
C15	0.0751 (11)	0.0751 (12)	0.1001 (12)	0.0149 (9)	0.0180 (9)	−0.0255 (10)
C16	0.0918 (14)	0.0594 (12)	0.1306 (16)	0.0150 (10)	0.0123 (12)	−0.0217 (11)
C17	0.0942 (13)	0.0550 (10)	0.1092 (13)	0.0036 (9)	0.0192 (10)	0.0030 (9)
C18	0.0743 (10)	0.0532 (9)	0.0796 (9)	0.0048 (7)	0.0198 (8)	0.0023 (7)
C19	0.0518 (8)	0.0539 (8)	0.0613 (7)	0.0067 (6)	0.0095 (6)	−0.0031 (6)
C20	0.0551 (8)	0.0685 (10)	0.0689 (8)	0.0096 (7)	0.0115 (6)	−0.0128 (7)
O11	0.0950 (8)	0.0586 (6)	0.0556 (5)	−0.0139 (6)	0.0134 (5)	−0.0038 (4)
N11	0.0614 (7)	0.0716 (8)	0.0549 (6)	−0.0077 (6)	0.0186 (5)	−0.0020 (6)
N12	0.0598 (7)	0.0743 (8)	0.0563 (6)	−0.0110 (6)	0.0149 (5)	−0.0050 (6)
C21	0.0629 (8)	0.0538 (8)	0.0543 (7)	−0.0084 (6)	0.0191 (6)	−0.0030 (6)
C22	0.0616 (8)	0.0542 (8)	0.0567 (7)	0.0013 (7)	0.0125 (6)	0.0035 (6)
C23	0.0585 (8)	0.0505 (8)	0.0638 (7)	0.0006 (6)	0.0146 (6)	−0.0032 (6)
C24	0.0583 (8)	0.0609 (9)	0.0516 (6)	−0.0058 (7)	0.0190 (6)	−0.0007 (6)
C25	0.0958 (12)	0.0601 (9)	0.0555 (7)	−0.0102 (8)	0.0199 (7)	0.0108 (7)
C26	0.1015 (12)	0.0518 (9)	0.0632 (8)	−0.0140 (8)	0.0215 (8)	0.0039 (7)
C31	0.0500 (8)	0.0912 (12)	0.0538 (7)	−0.0130 (7)	0.0127 (6)	−0.0054 (7)
C32	0.0581 (9)	0.1153 (14)	0.0648 (8)	−0.0132 (9)	0.0095 (7)	0.0071 (9)
C33	0.0673 (11)	0.171 (2)	0.0614 (9)	−0.0193 (13)	0.0040 (8)	0.0106 (12)
C34	0.0623 (10)	0.187 (2)	0.0624 (10)	−0.0302 (13)	0.0142 (8)	−0.0293 (13)
C35	0.0665 (12)	0.144 (2)	0.1131 (15)	−0.0354 (13)	0.0348 (11)	−0.0668 (14)
C36	0.0790 (14)	0.1076 (18)	0.160 (2)	−0.0263 (12)	0.0424 (14)	−0.0554 (16)
C37	0.0806 (13)	0.0863 (14)	0.1322 (16)	−0.0141 (10)	0.0253 (11)	−0.0219 (12)
C38	0.0669 (10)	0.0848 (12)	0.0875 (10)	−0.0135 (9)	0.0158 (8)	−0.0152 (9)
C39	0.0472 (8)	0.0973 (12)	0.0653 (8)	−0.0144 (8)	0.0172 (6)	−0.0201 (8)
C40	0.0526 (9)	0.1284 (16)	0.0755 (10)	−0.0249 (10)	0.0214 (7)	−0.0365 (11)

### Geometric parameters (Å, °)

**Table:**

O1—C1	1.3564 (14)	O11—C21	1.3539 (14)
O1—H1	0.8200	O11—H11	0.8200
N1—N2	1.2573 (13)	N11—N12	1.2566 (15)
N1—C4	1.4204 (15)	N11—C24	1.4192 (15)
N2—C11	1.4286 (15)	N12—C31	1.4223 (16)
C1—C6	1.3811 (17)	C21—C26	1.3785 (18)
C1—C2	1.3840 (18)	C21—C22	1.3811 (17)
C2—C3	1.3776 (17)	C22—C23	1.3746 (17)
C2—H2	0.9300	C22—H22	0.9300
C3—C4	1.3872 (16)	C23—C24	1.3879 (17)
C3—H3	0.9300	C23—H23	0.9300
C4—C5	1.3746 (17)	C24—C25	1.3774 (18)
C5—C6	1.3747 (17)	C25—C26	1.3793 (19)
C5—H5	0.9300	C25—H25	0.9300
C6—H6	0.9300	C26—H26	0.9300
C11—C12	1.3670 (17)	C31—C32	1.367 (2)
C11—C19	1.4091 (17)	C31—C39	1.418 (2)
C12—C13	1.3995 (18)	C32—C33	1.406 (2)
C12—H12	0.9300	C32—H32	0.9300
C13—C14	1.364 (2)	C33—C34	1.356 (3)
C13—H13	0.9300	C33—H33	0.9300
C14—C20	1.405 (2)	C34—C40	1.412 (3)
C14—H14	0.9300	C34—H34	0.9300
C15—C16	1.347 (2)	C35—C36	1.343 (3)
C15—C20	1.407 (2)	C35—C40	1.403 (3)
C15—H15	0.9300	C35—H35	0.9300
C16—C17	1.397 (2)	C36—C37	1.399 (3)
C16—H16	0.9300	C36—H36	0.9300
C17—C18	1.351 (2)	C37—C38	1.356 (2)
C17—H17	0.9300	C37—H37	0.9300
C18—C19	1.4189 (18)	C38—C39	1.411 (2)
C18—H18	0.9300	C38—H38	0.9300
C19—C20	1.4239 (18)	C39—C40	1.4265 (19)
			
C1—O1—H1	109.5	C21—O11—H11	109.5
N2—N1—C4	114.33 (10)	N12—N11—C24	114.97 (11)
N1—N2—C11	114.41 (10)	N11—N12—C31	115.02 (12)
O1—C1—C6	116.94 (12)	O11—C21—C26	116.91 (12)
O1—C1—C2	123.18 (12)	O11—C21—C22	123.29 (12)
C6—C1—C2	119.89 (12)	C26—C21—C22	119.80 (12)
C3—C2—C1	119.97 (12)	C23—C22—C21	120.51 (12)
C3—C2—H2	120.0	C23—C22—H22	119.7
C1—C2—H2	120.0	C21—C22—H22	119.7
C2—C3—C4	119.92 (12)	C22—C23—C24	119.74 (13)
C2—C3—H3	120.0	C22—C23—H23	120.1
C4—C3—H3	120.0	C24—C23—H23	120.1
C5—C4—C3	119.79 (12)	C25—C24—C23	119.61 (12)
C5—C4—N1	116.26 (11)	C25—C24—N11	116.85 (12)
C3—C4—N1	123.89 (12)	C23—C24—N11	123.53 (12)
C4—C5—C6	120.45 (12)	C24—C25—C26	120.53 (13)
C4—C5—H5	119.8	C24—C25—H25	119.7
C6—C5—H5	119.8	C26—C25—H25	119.7
C5—C6—C1	119.95 (13)	C21—C26—C25	119.81 (14)
C5—C6—H6	120.0	C21—C26—H26	120.1
C1—C6—H6	120.0	C25—C26—H26	120.1
C12—C11—C19	121.41 (12)	C32—C31—C39	121.16 (14)
C12—C11—N2	123.22 (12)	C32—C31—N12	123.57 (15)
C19—C11—N2	115.28 (11)	C39—C31—N12	115.26 (13)
C11—C12—C13	120.13 (14)	C31—C32—C33	119.80 (18)
C11—C12—H12	119.9	C31—C32—H32	120.1
C13—C12—H12	119.9	C33—C32—H32	120.1
C14—C13—C12	120.03 (14)	C34—C33—C32	120.48 (19)
C14—C13—H13	120.0	C34—C33—H33	119.8
C12—C13—H13	120.0	C32—C33—H33	119.8
C13—C14—C20	121.25 (14)	C33—C34—C40	121.64 (17)
C13—C14—H14	119.4	C33—C34—H34	119.2
C20—C14—H14	119.4	C40—C34—H34	119.2
C16—C15—C20	121.95 (17)	C36—C35—C40	120.91 (19)
C16—C15—H15	119.0	C36—C35—H35	119.5
C20—C15—H15	119.0	C40—C35—H35	119.5
C15—C16—C17	119.73 (16)	C35—C36—C37	120.5 (2)
C15—C16—H16	120.1	C35—C36—H36	119.8
C17—C16—H16	120.1	C37—C36—H36	119.8
C18—C17—C16	121.21 (17)	C38—C37—C36	120.6 (2)
C18—C17—H17	119.4	C38—C37—H37	119.7
C16—C17—H17	119.4	C36—C37—H37	119.7
C17—C18—C19	120.45 (15)	C37—C38—C39	121.00 (17)
C17—C18—H18	119.8	C37—C38—H38	119.5
C19—C18—H18	119.8	C39—C38—H38	119.5
C11—C19—C18	123.48 (12)	C38—C39—C31	123.82 (13)
C11—C19—C20	118.05 (13)	C38—C39—C40	117.58 (16)
C18—C19—C20	118.48 (13)	C31—C39—C40	118.59 (16)
C14—C20—C15	122.78 (15)	C35—C40—C34	122.30 (18)
C14—C20—C19	119.03 (13)	C35—C40—C39	119.44 (19)
C15—C20—C19	118.17 (15)	C34—C40—C39	118.26 (18)
			
C4—N1—N2—C11	180.00 (10)	C24—N11—N12—C31	−179.16 (11)
O1—C1—C2—C3	179.58 (13)	O11—C21—C22—C23	179.57 (12)
C6—C1—C2—C3	−0.7 (2)	C26—C21—C22—C23	−0.6 (2)
C1—C2—C3—C4	−0.4 (2)	C21—C22—C23—C24	0.8 (2)
C2—C3—C4—C5	0.21 (19)	C22—C23—C24—C25	−0.1 (2)
C2—C3—C4—N1	−176.74 (12)	C22—C23—C24—N11	178.54 (12)
N2—N1—C4—C5	166.80 (11)	N12—N11—C24—C25	−167.83 (13)
N2—N1—C4—C3	−16.15 (17)	N12—N11—C24—C23	13.54 (19)
C3—C4—C5—C6	1.1 (2)	C23—C24—C25—C26	−0.9 (2)
N1—C4—C5—C6	178.26 (12)	N11—C24—C25—C26	−179.57 (13)
C4—C5—C6—C1	−2.2 (2)	O11—C21—C26—C25	179.50 (13)
O1—C1—C6—C5	−178.27 (12)	C22—C21—C26—C25	−0.3 (2)
C2—C1—C6—C5	2.0 (2)	C24—C25—C26—C21	1.1 (2)
N1—N2—C11—C12	26.47 (17)	N11—N12—C31—C32	−19.79 (19)
N1—N2—C11—C19	−156.77 (11)	N11—N12—C31—C39	161.81 (12)
C19—C11—C12—C13	3.6 (2)	C39—C31—C32—C33	−3.1 (2)
N2—C11—C12—C13	−179.85 (12)	N12—C31—C32—C33	178.58 (13)
C11—C12—C13—C14	−1.2 (2)	C31—C32—C33—C34	1.5 (3)
C12—C13—C14—C20	−1.6 (2)	C32—C33—C34—C40	0.4 (3)
C20—C15—C16—C17	−0.3 (3)	C40—C35—C36—C37	0.4 (3)
C15—C16—C17—C18	−0.3 (3)	C35—C36—C37—C38	−0.7 (3)
C16—C17—C18—C19	0.1 (2)	C36—C37—C38—C39	0.7 (3)
C12—C11—C19—C18	176.81 (13)	C37—C38—C39—C31	−179.74 (15)
N2—C11—C19—C18	−0.01 (18)	C37—C38—C39—C40	−0.4 (2)
C12—C11—C19—C20	−3.18 (19)	C32—C31—C39—C38	−177.83 (14)
N2—C11—C19—C20	180.00 (11)	N12—C31—C39—C38	0.6 (2)
C17—C18—C19—C11	−179.40 (14)	C32—C31—C39—C40	2.8 (2)
C17—C18—C19—C20	0.6 (2)	N12—C31—C39—C40	−178.75 (12)
C13—C14—C20—C15	−176.48 (15)	C36—C35—C40—C34	−179.71 (18)
C13—C14—C20—C19	1.9 (2)	C36—C35—C40—C39	−0.1 (3)
C16—C15—C20—C14	179.36 (16)	C33—C34—C40—C35	178.93 (17)
C16—C15—C20—C19	1.0 (2)	C33—C34—C40—C39	−0.7 (3)
C11—C19—C20—C14	0.44 (19)	C38—C39—C40—C35	0.1 (2)
C18—C19—C20—C14	−179.55 (13)	C31—C39—C40—C35	179.49 (14)
C11—C19—C20—C15	178.90 (13)	C38—C39—C40—C34	179.71 (14)
C18—C19—C20—C15	−1.09 (19)	C31—C39—C40—C34	−0.9 (2)

### Hydrogen-bond geometry (Å, °)

**Table:**

*D*—H···*A*	*D*—H	H···*A*	*D*···*A*	*D*—H···*A*
O1—H1···N11	0.82	2.03	2.8380 (15)	167
O11—H11···N1^i^	0.82	2.04	2.8485 (15)	170

Symmetry codes: (i) *x*, *y*, *z*+1.
